# Antitumor effect of proanthocyanidin induced apoptosis in human colorectal cancer (HT-29) cells and its molecular docking studies

**DOI:** 10.1186/s13065-019-0525-7

**Published:** 2019-02-04

**Authors:** Mani Suganya, Balasubramanian Mythili Gnanamangai, Balasubramani Ravindran, Soon Woong Chang, Arokiyaraj Selvaraj, Chandramohan Govindasamy, Mohamed Farouk Elsadek, Ponnusamy Ponmurugan

**Affiliations:** 1 Department of Biotechnology, K. S. Rangasamy College of Technology, Tiruchengode, Tamil Nadu 637215 India; 2 Department of Environmental Energy and Engineering, Kyonggi University, Youngtong-Gu, Suwon, 16227 South Korea; 3 Department of Food Science and Biotechnology, Sejong University, Seoul, Republic of Korea; 4 Department of Community Health Sciences, College of Applied Medical Sciences, King Saud University, P.O. Box 10219, Riyadh, 11433 Saudi Arabia; 5 Department of Botany, Bharathiar University, Coimbatore, Tamil Nadu 641 046 India

**Keywords:** Proanthocyanidin, AO/EtBr, Cell cycle arrest, Molecular docking

## Abstract

Proanthocyanidin (PAC) is a promising compound that has displayed its potent antineoplastic properties with a specific intrinsic pathway. This precise us to explore the phyto-preventive effect of PAC against colon cancer (HT-29). The results showed that PAC inhibited the cell growth and GI_50_ value was found to be 6.25 μM for 24 h exposure, when correlated to the normal cell line does not have toxicity was noticed. The linguistic differences, similarly membrane blebbing, cell shrinkage fragmented nuclear bodies and mitochondrial membrane were observed in AO/EtBr and DAPI staining. The features of regular mechanical apoptotic characterization was analyzed by DNA fragmentation. The cell cycle arrest at G2/M phases was detected using FACS analysis. The early and late apoptotic cells were observed by using Annexin V/PI staining. The ligand–protein interaction and docking studies were performed using Schrodinger’s software. The QPLD analysis of docking studies revealed that PAC exhibited better binding affinity of − 5.23, − 5.17 and − 4.43, − 4.47 kcal/mol against BCL-XL, CDK2 and were compared with 5-FU respectively, which significantly reveals the anticancerous activity of Proanthocyanidin compound. Thus, the PAC compound provides future application of therapeutic option in the treatment of colon cancers
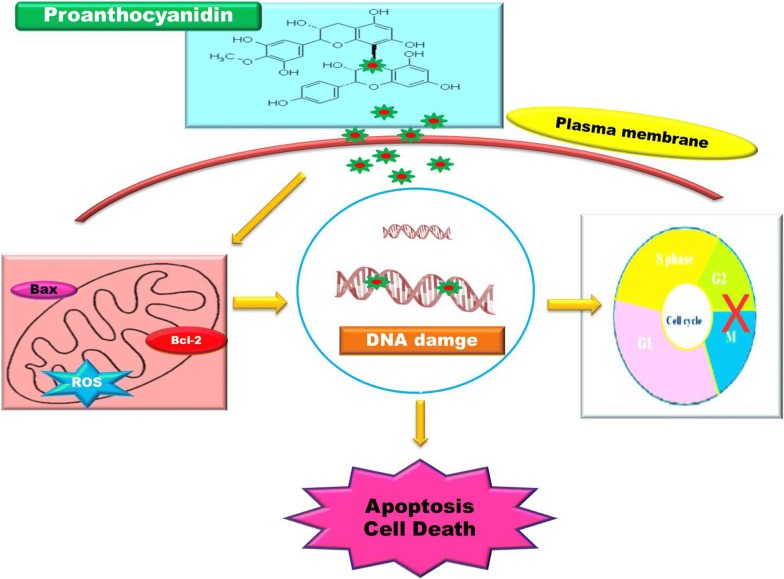
.

## Introduction

Colorectal cancer (CRC) is the highest prevailing destructive tumor mortality in worldwide [1]. CRC is treated with surgery, radiation and chemotherapy, depending on the tumor site and disease stage. These chemotherapy drugs are formulated to disrupt tumor cell microtubule signals and hyperproliferative status of these cells has been inhibiting the cell-cycle arrest and subsequently inducing cell death [2]. The cell cycle progression of G1/S and G2/M phases derived from cyclin-dependent kinases (CDKs). The CDKs proteins binding with exact cyclins through sequential activation of cell cycle progression. Cell cycle regulating protein cyclin A is fixed to CDK2 or CDC2 proteins, a crucial aspect in rectifying the S phase transformation.

Hence the several chemotherapeutic phytomedicines, flavonoids have been a comprehensive part in preventing cell generation and transition. Edible vegetables, grains and grass are a fabulous source of flavonoids. These flavonoids have acquired a developing consideration in the field of pharmacology due to their assorted therapeutic effects. The access to phyto-medicine had been adopted, strongly in the therapy for many groups of tumor [3, 4], which produced better chemopreventive effects when they are derived from natural sources. The low concentration of phyto-medicine compound displays successful treatment for treating malignancy cells without influencing typical cells.

Proanthocyanidin (PAC) isolated from the *Vitis vinifera*, is a well-known phytochemical due to their numerous biomedical application, containing oxidation inhibitor, anti-irritation, neurological, low blood sugar, cardiac disorders, antitumor, etc. [5, 6]. Generally, PAC may prevent a sequence of signal interchange mechanisms identical to cancer causing and behaves as an appropriate receiver of tyrosine enzyme and proteasome [7]. When treated with PAC, a polyphenolic compound, could specifically bind to nucleic acids, and prompt the damage of DNA in tumor cells by controlling the function of DNA topoisomerase and finally affects cell death [8], that is related among the DNA bruise response (DBR). Hence, DBR causes cell suicide, namely caspase-mediated cell death, whereas DNA loss may not be effectively rectified [9]. The DBR frequently generates autophagic cell destruction in tumor cells. A huge number of studies exposed that (−)-Epigallocatechin gallate (EGCG) plus curcumin shown impressive malignant tumor and therapeutic actions of less toxic following different types of carcinoma over the diverse processes [10, 11]. Hence, this study is aimed to investigate whether the natural compound PAC could exert therapeutic effects, in particular to colon cancer (HT-29) cells.

Computational supported drug screening techniques is frequently used to forecast through an extensive study of ability, this structure binding with the relevant targeted small molecule ligands. Moreover, that can be selected by analyzing the interaction between phytochemicals [12]. The phenomenal research was checked to study the efficiency of the PAC compound to generate more repressive action on HT-29 cells in vitro and in silico analyses.

## Materials and methods

### Materials

Proanthocyanidin (PAC) compound was acquired from Sigma-Aldrich (St. Louis, MO, USA). 3-(4,5-dimethylthiazol-2-yl)-2,5 diphenyltetrazolium bromide (MTT), Propidium iodide (PI), Dulbecco modified eagle’s medium (DMEM), Fetal Bovine Serum (FBS), Penicillin, and Streptomycin, Dimethyl sulfoxide (DMSO), DNA extraction kit was obtained from (Hi-media, Chennai). All the chemicals and reagents utilized were should be more specific.

### Cell culture

Human colorectal cancer (HT-29) and normal cell line FHC (human fetal normal colonic mucosa) was procured from National Center for Cell Sciences (NCCS), Pune, India. This cell line was grown in RPMI-1640 (Gibco-BRL) medium containing (10% v/v) warm-suspended fetal bovine serum (Gibco-BRL) additionally 2 mM-glutamine (Sigma Chemical); Penicillin (100 mg/mL); Streptomycin (100 mg/mL). These supplemented media are referred to as complete media or growth media and routinely cultured at 37 °C in a 5% CO_2_ water-saturated atmosphere. Cells were passaged and subculture to 90% confluence with 0.2% trypsin (w/v) every 2–3 days.

### Cell growth inhibitory assay

The survival of treated carcinoma cells was checked through MTT assay. Briefly, cells were patched in 96-well dishes at a viscosity of 4 × 10^3^ cells/mL inside 100 µL supplement and permitted to adhere overnight. After incubation, increasing concentrations (1.56–50 μM) of the PAC was affixed and grown for 24 h. Then 50 µL of MTT (3 mg/mL in PBS) was covered for 4 h at 37 °C. The unbounded solution was detached likewise each wells were added 100 µL of DMSO. The optical density was detected at 570 nm by using small SYNERGY HTX SILFA multi-mode reader (BioTek, USA). The irregular shapes were visualized under bright-field inverted phase contrast light microscope (Nikon, Japan) at 400× magnification.

### Acridine orange and ethidium bromide staining

Apoptosis was determined by the detection of nuclear morphology using AO/EtBr staining methodology and it is based on the difference in membrane integrity between apoptosis and necrosis. The sterile coverslip was placed in 6-well tissue culture plates while cells were inoculated at a concentration of 4 × 10^3^ cells/mL. Following incubation, the medium was evacuated and replenished with medium containing FBS (10%) along with the cells processed through PAC compound with 24 h. The cells were disinfected from PBS and rigid with 4% P-formaldehyde for 5 min at 4 °C. Further, cell culture was stained with an AO/EtBr solution (100 µg/mL AO and 100 µg/mL EtBr in PBS) proceeding in dark condition. This suspension was viewed under a fluorescence emitting microscope (Nikon, Eclipse 600, Japan) 400× amplification at 510–590 nm.

### DAPI staining

The DAPI is a blue fluorescent dye which is sensitive to chromatins and very less toxic to cells, it measured to observe the nuclei changes in apoptotic cells. HT-29 cells were patched in 6-well dishes and maintained at 37 °C along 5% CO_2_ and incubated for 24 h. The cells were prepared at GI_50_ absorptions of (3.12 µM, 6.25 µM and 12.5 µM) for 24 h. Then the cells were disinfected using PBS and treated with 4% P-formaldehyde for 15 min, it was then permeabilized with 0.1% Triton X-100 and stained with DAPI (1 mg/mL) for 10 min. The stained cells were pictured using a fluorescence microscope with the suitable excitation filter.

### DNA disintegration analysis

DNA disruption research was conducted using an agarose gel electrophoresis method. In brief, appropriate GI_50_ absorption of PAC activated cells were grown in 10 cm culture plate. Together connected and detached cells were unstained with ice-cold PBS. The cells were thawed in 500 µL of lysis buffer (0.5% Triton X-100, 10 mM EDTA, 10 mM Tris–HCl, pH 8.0 and 2 mg/mL proteinase K) and were treated for 3 h at 55 °C, additionally RNAse A (Amrsco, Solon, OH) was supplemented for 3 h. DNA was extracted with saturated phenol: chloroform (1:1), accelerated using ethanol precipitation along with Tris/EDTA buffer (10 mM Tris–HCl, pH 8.0, and 1 mM EDTA). The detected DNA was visualized in 2% agarose gel electrophoresis, incorporated with ethidium bromide (0.5%), and were observed using UV-trans illuminator system (Bio-Rad).

### Analysis of cell cycle progression

To regulate the consequence of PAC and their promoting apoptosis on the cell division in HT-29 cell line, the cells were exposed to the pre-determined GI_50_ concentrations of test compound were measured with 24 h untreated control cells (considered with drug free media). Cells were harvested and trypsinized, followed by rinsing with PBS and extended in binding buffer. The cells were labelled with propidium iodide in PBS containing RNAse (0.1 mg/mL). The labelled cells were examined under FACS Calibur (Becton–Dickinson, San Jose, CA) flow cytometer. Cell division and apoptotic ratios in Sub-G0, G1/S and G2/M phase were consequently determined using BD Cell Quest Pro Software (Version 5.1).

### In silico study

#### Desktop configuration

The high performance GPU operated with Cent OS V6.6 were used to study molecular modeling with the help of the Linux operating platform. Computer stipulations of the HPC GPU-Super micro Intel Xeon E5-1620 v3 series of specification 32 GB DDR4-2133 ECC RDIMM of RAM and 2 GB Graphics card of NVIDIA Quadro K620 with 4-core processor. The specific docking software used is a commercial version of the Schrodinger software package, LLC, New York, NY 2015.

#### Protein preparation

For the analysis of docking simulation, the protein complex was retrieved from protein data bank (PDB) as shown in (Table 3). The receptor protein structure for the docking simulation was pre-processed using the Protein Preparation Wizard module in Schrodinger software [13]. The protein residues His, Asp and Glu residue gets into protonated states, when H-atoms are added along with hydrogens on thiols and hydroxyls and were sampled to H-bond optimize network. This Schrodinger provides missing side chain alignment of each residue using the build interface incorporation. The OPLS-2005 force field used to optimize the state subsequently for the minimization process with an Impact Refinement module to condense steric clashes that may exist in the structures. When the average root mean square value of non-hydrogen atoms reached the 0.3 Å, the minimization was terminated [14].

#### Ligand preparation

Proanthocyanidin ligand was prepared for structure optimization and conformer generation using LigPrep [15]. The 2D companion into 3D conformer was converted in all the compounds. The optimized energy conformers of ligand were obtained by the application of OPLS force field. Neutralization of charge groups, addition of implicit hydrogen atoms, tautomerization, generation of various ionization, and chiralities states of the ligand molecule were the prior steps taken before the energy minimization process of ligand structures [14].

#### Quantum polarized ligand docking

The partial charges on the ligand atoms was enhanced by quantum-polarized ligand docking (QPLD) in a Glide docking run and replacing them with charges received from quantum automatic prediction. In QPLD using Glide, the chosen ligand was docked and charges on the ligand elicited by the protein are studied, the best ligand interactions are re-docked. Molecular mechanics and quantum mechanics (QM/MM) (efficiency/velocity) combined option was provided by QPLD. The accuracy was enhanced with minimum accomplishing duration with the united QM/MM approach for the atomistic level prediction of binding energy and charge transition. Interaction between ligand and the binding site of protein was done by QM calculations of QPLD and the remaining protein region was calculated by MM force field [16].

The marginal partial charge cut-off value of 0.25, the vander Waal radii of receptor atoms were scaled with 2.00 Å to soften the potential for non-polar part of the receptor were selected. The charge calculation, selection of QM level is a tradeoff between accuracy and speed. The surface electrostatic potential energy is used for calculating partial charges by accurate and fast modes. Fast uses the B3LYP functional, ‘Quick’ self-consistent field (SCF) and 3–21G basis set of accuracy level. For the functional theory calculation in the QM region accurate uses B3LYP, ‘Ultrafine’ SCF accuracy level (iacc = 1, iacscf = 2) and the 6–31G⁄/LACVP⁄ basis set. Quantum mechanics cover the ligand and active site region and the remaining protein system comes under molecular mechanical mode.

#### Binding free energy calculation

The binding free energy calculation is applied to the docked complex using Molecular Mechanics Generalized Born Surface Area (MM-GBSA) path engaged through Prime 3. The solvation and entropy or polarizability assuming that, they do not perfectly account when the scoring functions may fail. OPLS-2005 modulation range and GBSA continuum solvent model are depleted to confirm the efficiency of docking score, whatever proved and establishment of the docking complex. The following equations were used to calculate the Binding energy [17].

1 $$ \Delta {\text{G}}_{\text{bind}} =  \Delta {\text{E }} + \Delta {\text{G}}_{\text{solv}} +  \Delta {\text{G}}_{\text{SA}} $$

2 $$ \Delta {\text{E }} = {\text{ E}}_{\text{complex}} - {\text{E}}_{\text{protein}} - {\text{E}}_{\text{ligand}} $$

The protein-inhibitor complex were minimized the energies of (E_complex_, E_protein_, and E_ligand_) respectively.

3 $$ \Delta {\text{G}}_{\text{solv}} = {\text{ G}}_{\text{solv}} \left( {\text{complex}} \right)  - {\text{ G}}_{\text{solv}} \left( {\text{protein}} \right)  - {\text{ G}}_{\text{solv}} \left( {\text{ligand}} \right) $$

where, ∆G_solv_ is generalized born electrostatic solvation energy. G_solv_ (complex), G_solv_ (protein) and G_solv_ (ligand) are the solvation complementary strength of ambiguous, protein and ligand respectively.

4 $$ \Delta {\text{G}}_{\text{SA}} = {\text{ G}}_{\text{SA}} \left( {\text{complex}} \right)  - {\text{ G}}_{\text{SA}} \left( {\text{protein}} \right)  - {\text{ G}}_{\text{SA}} \left( {\text{ligand}} \right) $$

where, ∆G_SA_ is the non-polar contribution to the solvation intensity receivable of the exterior field. G_SA_ (complex), G_SA_ (protein) and G_SA_ (ligand) are the surface energies of complex, protein and ligand respectively.

The GBSA continuum models were carried out for simulations. Gaussian surface is used instead of a van der Waals surface for better representation of solvent-accessible surface area with surface generalized Born (SGB) using by Prime model [18].

#### Statistical analysis

All the statistical calculation was carried through SPSS version 20.00 for Windows. The entire data were communicated as mean ± SE of triplicate independent analysis. One-way ANOVA with Dunnett’s post hoc test for multi-group comparisons (except the GI_50_ values which were estimated by nonlinear regression analysis using GraphPad Prism software, (Version 5.0). A p-value of 0.05 or less was examined as significant.

## Results and discussion

### Cell growth inhibitory assay

**Fig. 1:**
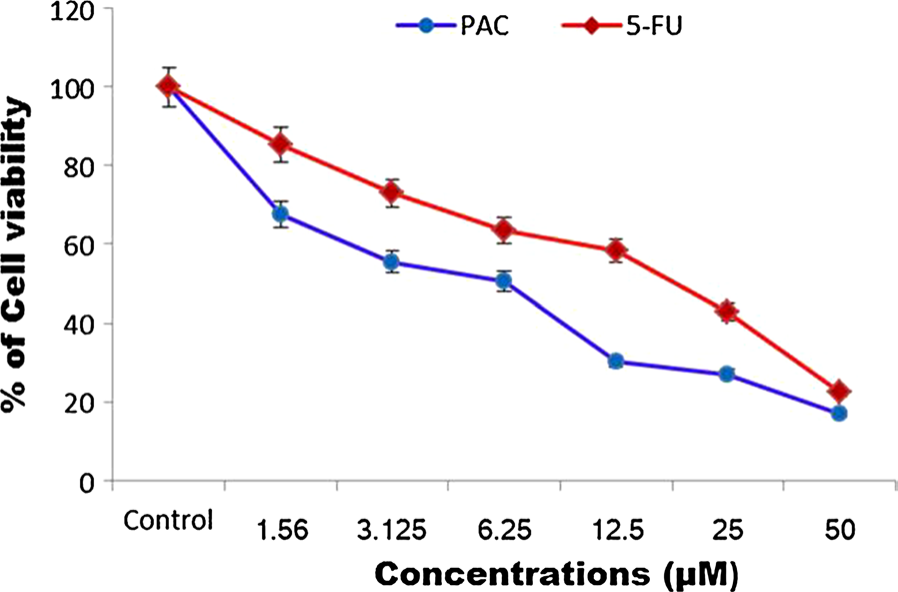
Cytotoxic effect of PAC exerts against Colon cancer (HT-29) cells. Cells were treated with indicated concentrations of PAC alone for 24 h. Cell viability was detected by MTT assay. The experiments were carried out in triplicate and each value represents mean ± SE

To determine the potential antitumor activity of PAC on HT-29 cells and fetal normal colonic mucosa (FHC) cells in increasing concentrations of (1.56–50 μM) for 24 h and different sensitivity of the cell lines are exhibited in each compound. Cell growth was evaluated by MTT assay. The PAC and 5-FU were treated in a dose-dependent manner to exhibit the growth restriction of HT-29 cells, wherein the percentage of cell viability was determined as 16.94 ± 0.78 and 22.38 ± 0.92 in a broad concentration range of 50 μM for 24 h respectively. However, treatment of PAC significantly enhanced the restrained growth with reduced growth inhibition called GI_50_values (55.38 ± 0.24, 50.65 ± 0.60 and 30.26 ± 0.12% cell viability respectively at the rate of 3.12, 6.25 and 12.5 μM). The GI_50_ value of the PAC in FHC cells was found to be 50 μM concentration have exhibited the maximum cytotoxic effect of 30% cells. Only the PAC compound decreased cell viability by more than 50% for HT-29 cell lines compared with untreated controls (Fig. 1). Therefore, these concentrations (3.12, 6.25 and 12.5 μM) of the PAC were used for later experiments. These values obviously proposed to the PAC possibly affect tumor cells (HT-29) than non-cancerous FHC cells. In this study, we tested anticancer therapeutic property of the PAC compound on Human Colon cancer cell line. EGCG and curcumin is well-known polyphenolic chemical structures, which are displaying the remarkable antineoplastic properties through various apoptotic functions [19]. When high dose of the compound is applied the anticancer efficiency is negotiating due to the nonspecific cytotoxicity with particular intrinsic cytotoxic potential [20]. The anticancer therapeutic effects of the PAC treatment are highly speculated in view of targeting properties in different molecules or pathways.

### Morphological observation

**Fig. 2:**
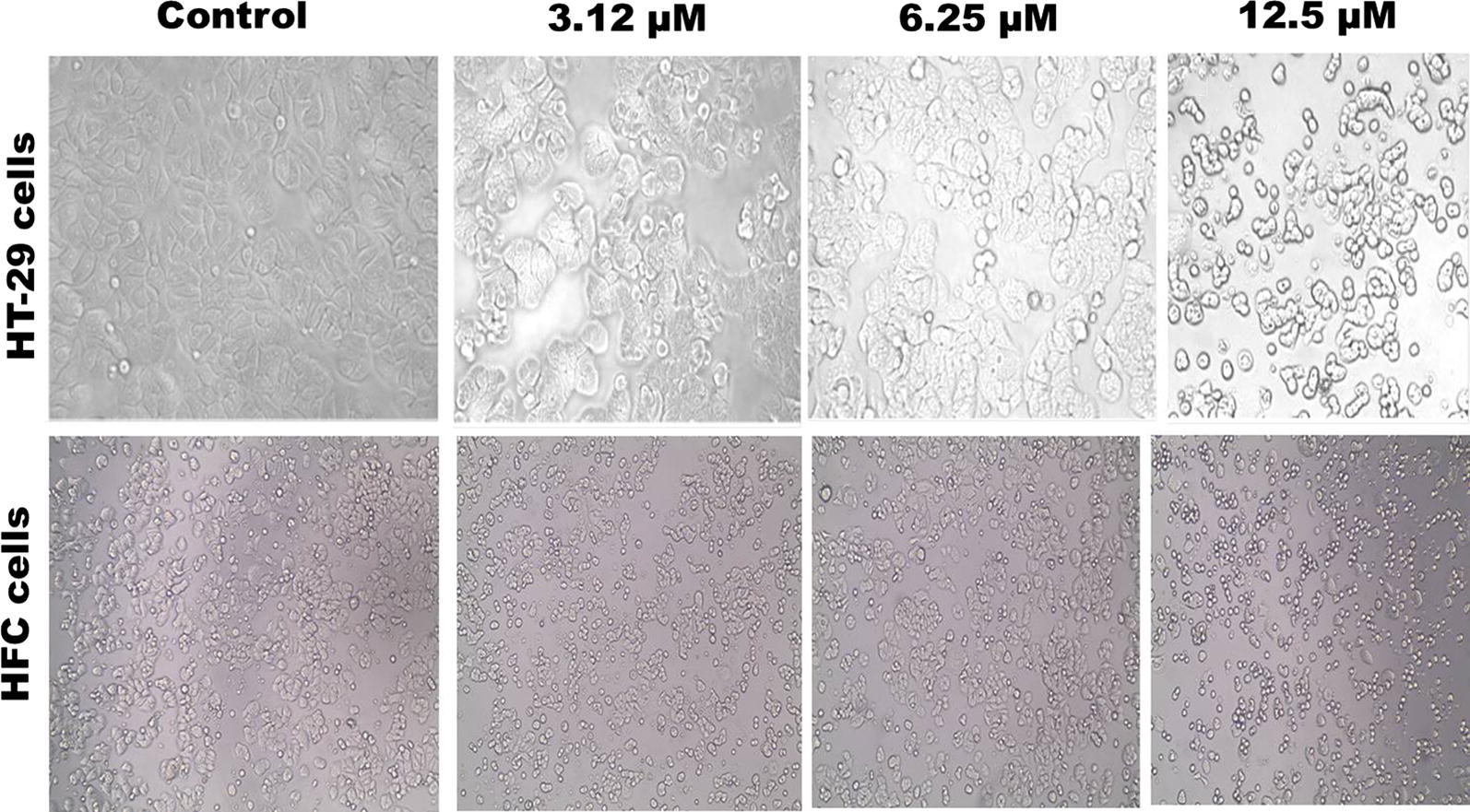
Light microscopic image of HT-29 cells treated with PAC for 24 h. Affected cells showed some morphological characteristic of apoptosis such as cell shrinkage and membrane blebbing at (×400) magnification

PAC compound induced apoptotic effects in HT-29 cells was visualized under an optical-inverted light microscopy. The cells treated with GI_50_ concentrations were chosen based on the MTT assay and incubated for 24 h. The polygonal and irregular shapes were exhibited in strongly attached mode. The original morphology with several nuclei was maintained by the untreated HT-29 cells and displayed normal, healthy round shape and a consistent size, with smooth cell membrane as shown in (Fig. 2). On the other hand compound treated HT-29 cells appeared smaller, less refracted, rounded, contraction, cellular blebbing and damaging interact with near cells. When compared to normal cells monolayer of the PAC treated cells (3.12, 6.25 and 12.5 μM) were found to be significantly destructed as compared to untreated cells. These emblematic shapes of biological differences in apoptotic cells extensively describes the apoptosis. The cells gradually became shrunken with the nuclear condensation, dissever chromatin, development of apoptotic body formation and chromatin condensation. The nucleus can be branched into nucleolytic pyknosis (primarily appear in apoptosis) [21]. Apoptosis surface morphology observed was very similar to the HT-29 cells, when treated with PAC. It was clearly differentiated with cellular morphological characteristics, which typically indicated on programmed cell death in cancer cells.

### AO/EtBr staining

**Fig. 3:**
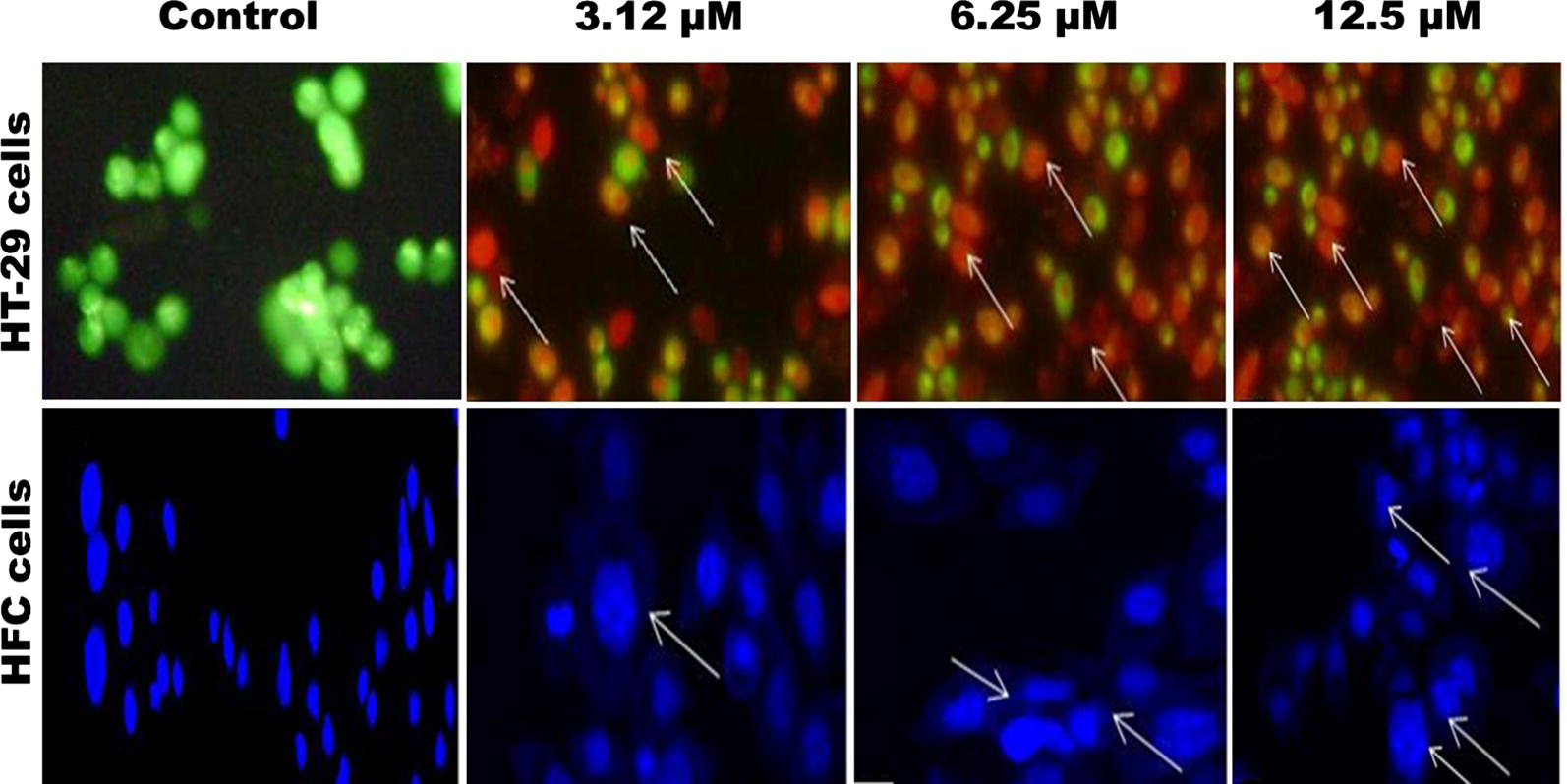
Nuclei morphological changes seen in HT-29 cells stained with AO/EtBr fluorescence microscopy. HT-29 cells were seeded in 6-well plates (4 × 10^3^ cells/mL) and treated with PAC compound with GI_50_ concentration respectively for 24 h at 37 °C

AO/EtBr and DAPI staining was used to assess the apoptosis and necrosis of cell death in HT-29 cells that are processed for GI_50_ concentrations of PAC 24 h, respective to cell growth inhibitory assay. While an untreated, HT-29 cells were maintained in growth media and marked from dual (AO/EtBr) and DAPI (Fig. 3). The superior noticeable apoptotic differences of PAC cured cells were seen in the cell loss, nuclear condensation, membrane blebbing fragmented nuclear bodies and mitochondrial membrane. The structural characteristic of HT-29 cells in apoptosis was displayed for 3.123, 6.25 and 12.5 μM of PAC using AO/EtBr and DAPI staining revealed better apoptosis. Acridine Orange (AO) stains strong cells, while Ethidium Bromide (EtBr) intercalates and stains double-stranded DNA of dead cells that have departed plasma membrane integrity. The nuclei of cells undergone apoptosis has conjugation with green, although the nuclei come out and disintegrated. Late apoptotic and necrotic cells appear orange and red, respectively [22].

### DNA fragmentation

**Fig. 4:**
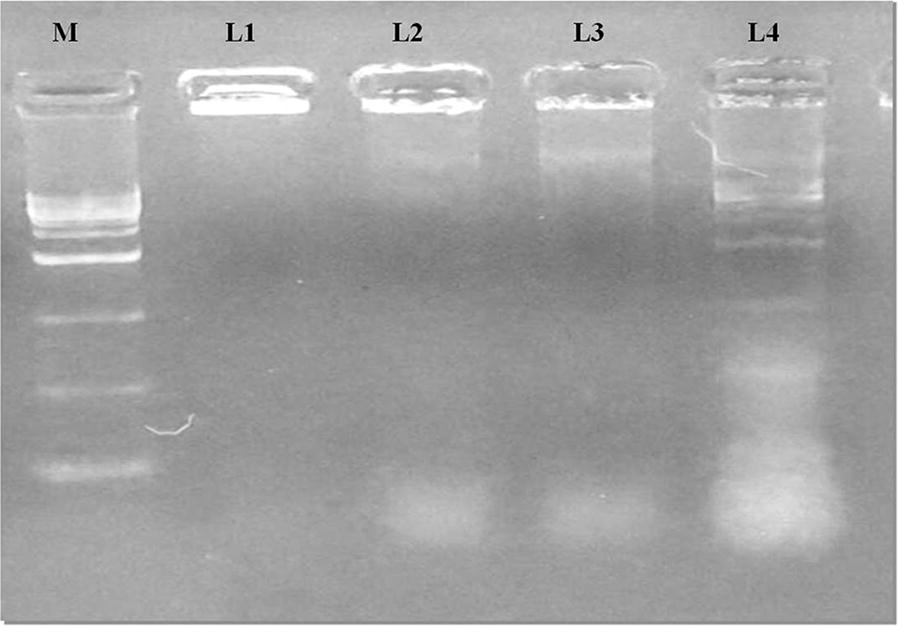
Analysis of DNA fragmentation induced by PAC in HT-29 cells for 24 h. Control cell lines incubated with DMEM medium alone. DNA fragmentation was assessed by 1.5% agarose gel electrophoresis and ethidium bromide staining and viewed under a UV transilluminator. Fragmented internucleosomal DNA appears as a ladder. “M” represent 1 bp DNA marker, (L1) Untreated, (L2) 3.12 µM, (L3) 6.25 µM and (L4) 12.5 µM

Genomic DNA fragmentation was done to elucidate the intrinsic apoptotic cell death. The nucleotide fragments were depicted under agarose gel electrophoresis with unique ladder composed at an interval of 180–200 base pairs. The presence of DNA fragmentation in HT-29 cells were considered with GI_50_ concentrations of PAC for 24 h. The DNA band was not observed in control (untreated) cells. An expansion in endonuclease to resolve the separated DNA ladder magnitude was detected with increasing concentration of PAC after 24 h, confirming the apoptosis. Interestingly, the DNA disintegration changes in the activated cells, with PAC were significantly high while linked to the lower concentration (Fig. 4). Our data corroborates with the DNA like pattern elucidated by curcumin and catechin compounds on HCT15, HCT116 and HepG2 cells which induced apoptosis [23]. The confirmed cell death via apoptosis by PAC was acquired and this compound may act through the apoptotic signaling and might have secured the anticancer effects.

### Analysis of cell cycle progression

**Fig. 5:**
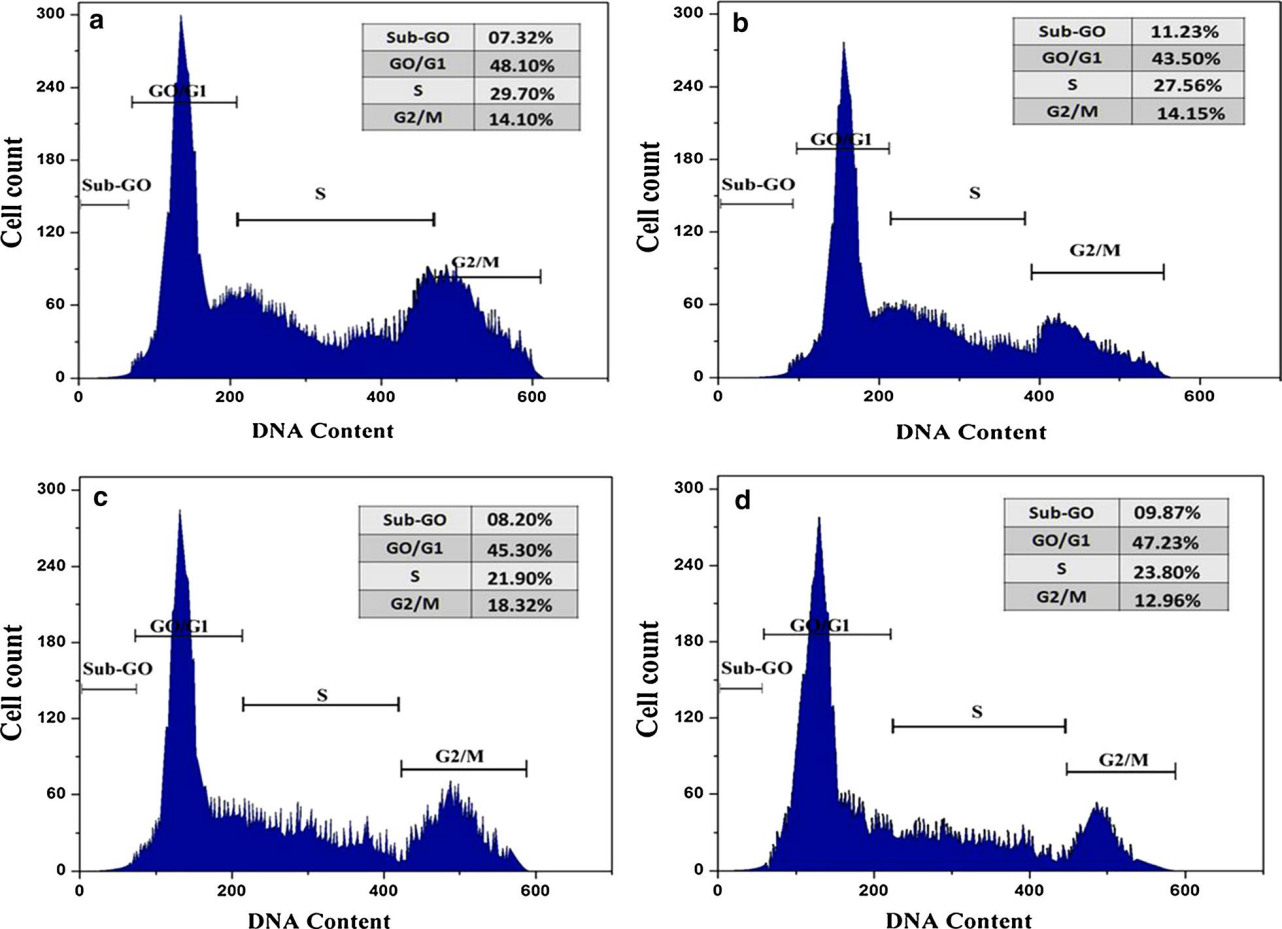
Cell cycle analysis of PAC induced apoptosis in HT29 cells by using Flow Cytometry. **a** Untreated cellular DNA was stained with PI and the distribution of the cells in G0/G1, S, and G2/M phase analyzed, **b** 3.12 µM, **c** 6.25 µM and **d** 12.5 µM

The cell division is a complex approach where cells obtained diverse, growth-controlling indicators that are combined and refined at different points known as checkpoints. The restricted cell division of HT-29 cells treated with PAC was evaluated by using flow cytometry. PAC treated at expanding concentration demonstrated in certain quantity of HT-29 cells at G1 phase with a reducing trend in those S phase and G2/M phase. Hence, perceived a significantly raise of cell cycle block in the G2/M phase with a reducing cell number in the S and G2/M phases at decrease concentrations of PAC (Fig. 5). The cell division was observed in G2/M phase; this information proposed that PAC compound significantly prompted apoptosis in the HT-29 cells. The anti-cancer has been considered as a targeted cell cycle progression for many years. The cell cycle arrest in A549 cells was significantly increased when treated with curcumin [10]. The cell cycle arrest was shown in G1 and S/G2 phase when treated with a combination of EGCG plus curcumin, where as affected the cyclin B1 along with cyclin D1 [24].

### Annexin V/PI

**Fig. 6:**
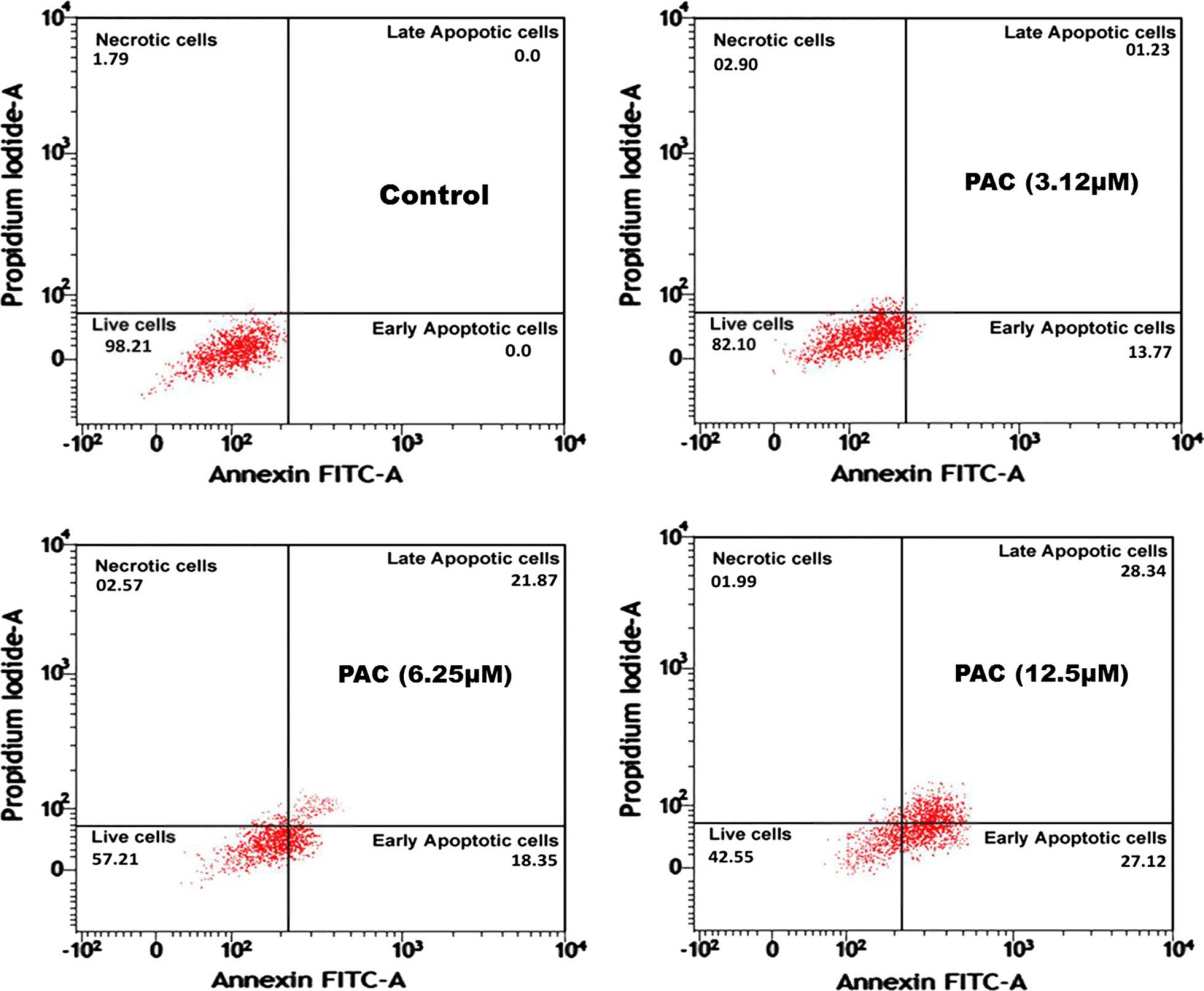
Treatment of PAC compound induces apoptosis in HT-29 cells were analyzed using Annexin V-FITC/PI double staining assay

Annexin V/PI assay was used to determine the apoptotic and necrotic cell population based on flow cytometry. Results from the number of cells entering initial and delayed apoptotic cells (13.77 and 1.23% in GI_50_ concentration of 3.12 µM; 18.35 and 21.87% in 6.25 µM concentration of PAC; 27.12 and 28.34% in 12.5 µM concentration of PAC respectively) and necrotic cells (2.90% in 3.12 µM concentration of PAC; 2.57% in 6.25 µM concentration of PAC and 1.99% in 12.5 µM concentration of PAC) related to control cells (Fig. 6). This data show a significant enhance in the proportion of early cell death in PAC dose-dependent manner. This outcome intimated that apoptosis induction contributes to the significantly killing of HT-29 cells by PAC compound. Phosphatidylserine (PS) externalization is an early indicator of apoptosis. FITC annexin V is frequently tested infusion with propidium iodide to regulate the initial apoptosis, forward to the damaged cell lamina integrity. Externalization of PS to the cell surface occurs in apoptotic cells. In comparing to the intrinsic and the extrinsic pamphlet of the plasma membrane has been transferred into phosphatidylserine (PS). Apoptotic cells due to the intact nature of membrane do not take up, hence PI can be differentiated from necrotic cells [25].

### QPLD analysis of docking scores

**Fig. 7:**
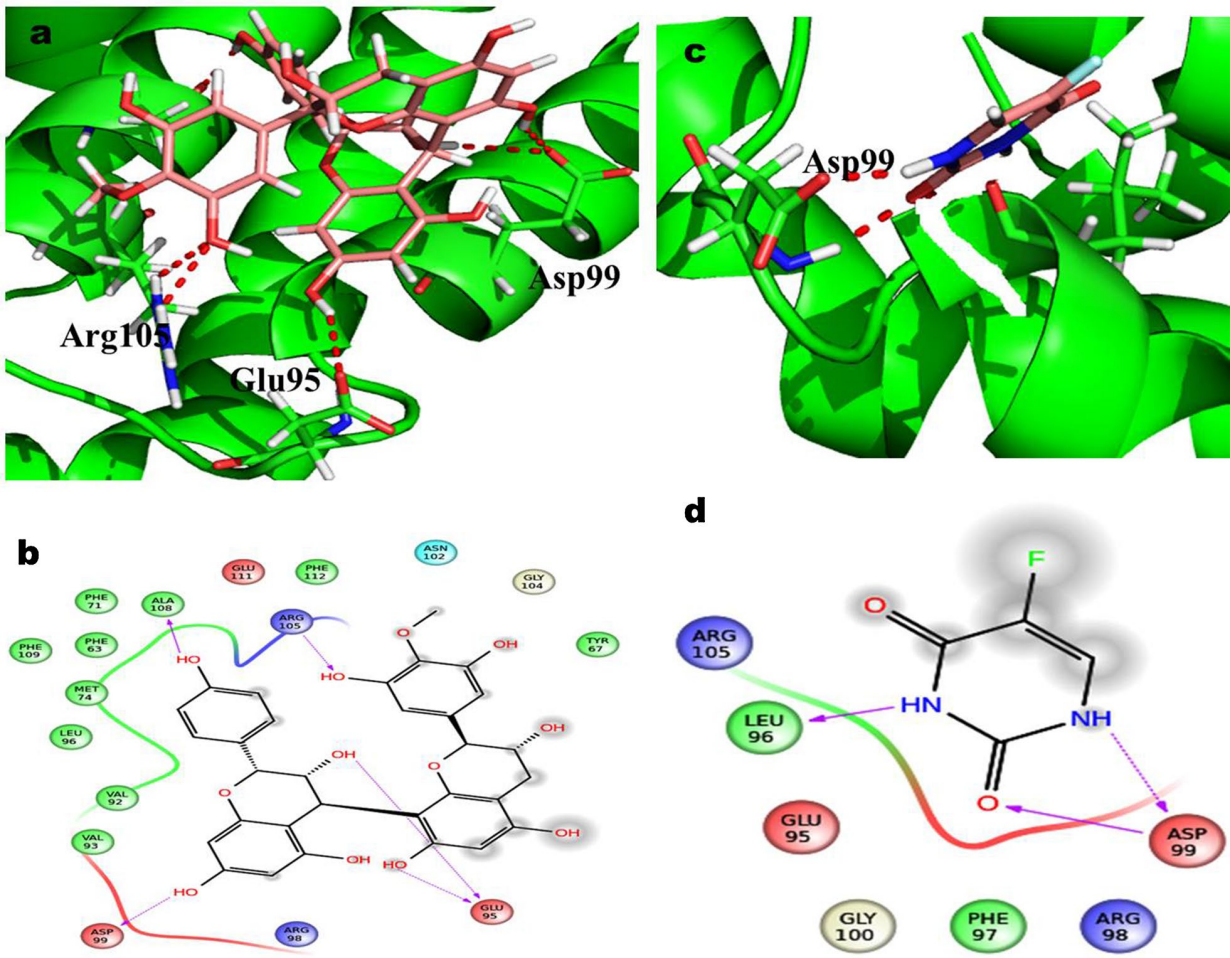
Glide XP docking interaction poses of proanthocyanidin (PAC) and 5-FU with BCL_2_ **a** PAC, **b** QPLD docking poses of PAC, **c** 5-FU and **d** QPLD docking poses of 5-FU

**Fig. 8:**
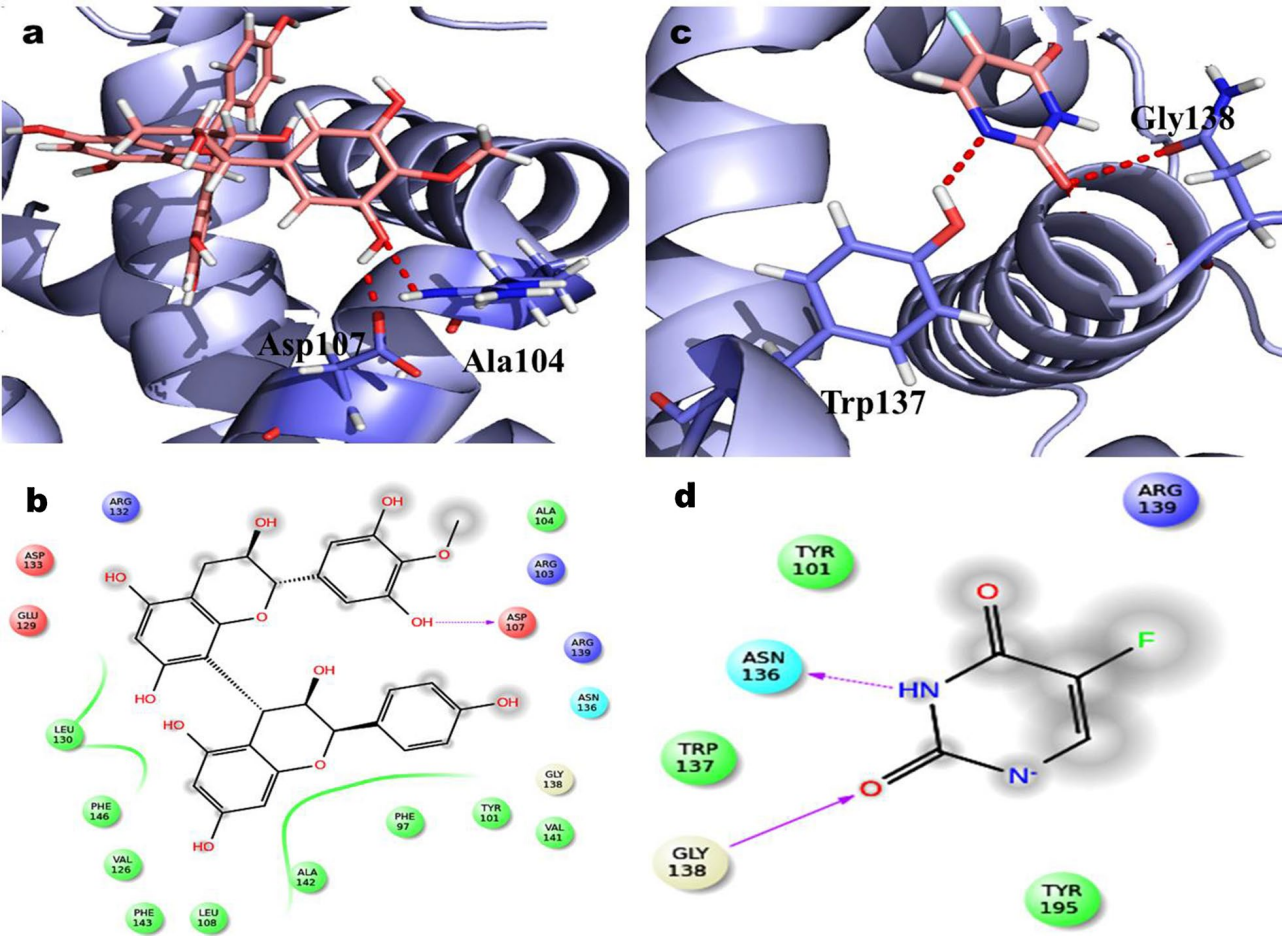
Glide XP docking interaction poses of proanthocyanidin (PAC) and 5-FU with BCL-XL **a** PAC, **b** QPLD docking poses of PAC, **c** 5-FU and **d** QPLD docking poses of 5-FU

The proanthocyanidin compounds (ligand) were docked with cell cycle (CDK2 and CDK4) and apoptotic (Bcl2, Bcl-XL) proteins against colon cancer applying the software of Schrodinger package. The QPLD docking interpretation of Proanthocyanidin displayed the five hydrogen bond interaction for essential amino acid of Arg105, Glu95 andAsp99 in targeted apoptotic protein BCL-2 complicated as shown in Fig. 7. The correspondingly 5-FU revealed that the dual hydrogen bond communication of Asp99 effective trash with BCL-2. The calculated docking score for Proanthocyanidin and 5-FU in QPLD was showing − 4.51 and − 3.72 kcal/mol, respectively. In QPLD approach, two different hydrogen bonds through Asp107 and Ala104 residue were observed (Fig. 8). However, in the case of 5-FU, two different hydrogen bond interaction with Trp1337 and Gly138 residues targeted apoptotic protein BCL-XL. The observed QPLD docking score were − 5.23 and − 4.43 kcal/mol in Proanthocyanidin and 5-FU.

**Fig. 9:**
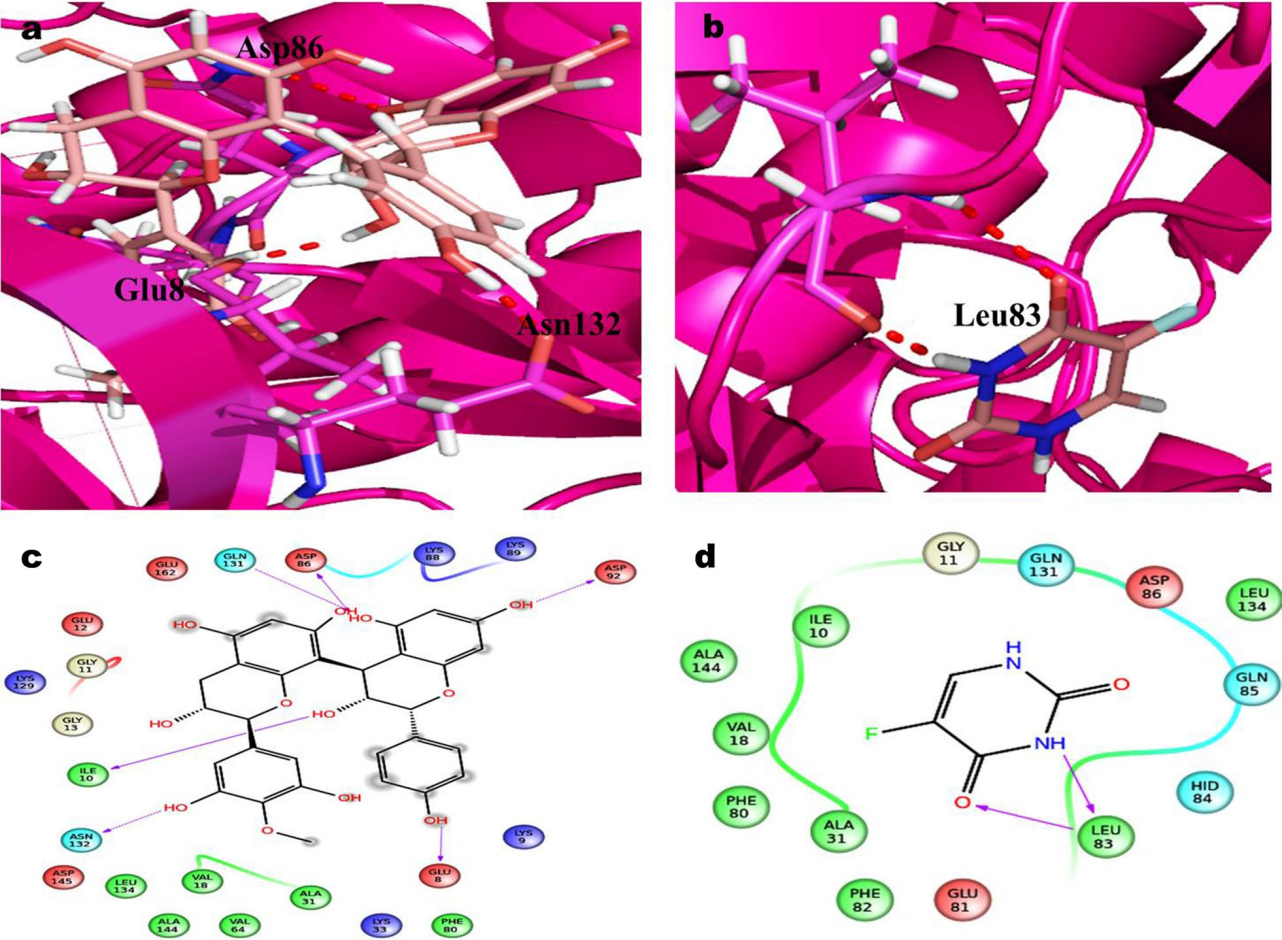
Glide XP docking interaction poses of Proanthocyanidin (PAC) and 5-FU with CDK2 **a** PAC, **b** QPLD docking poses of PAC, **c** 5-FU and **d** QPLD docking poses of 5-FU

**Fig. 10:**
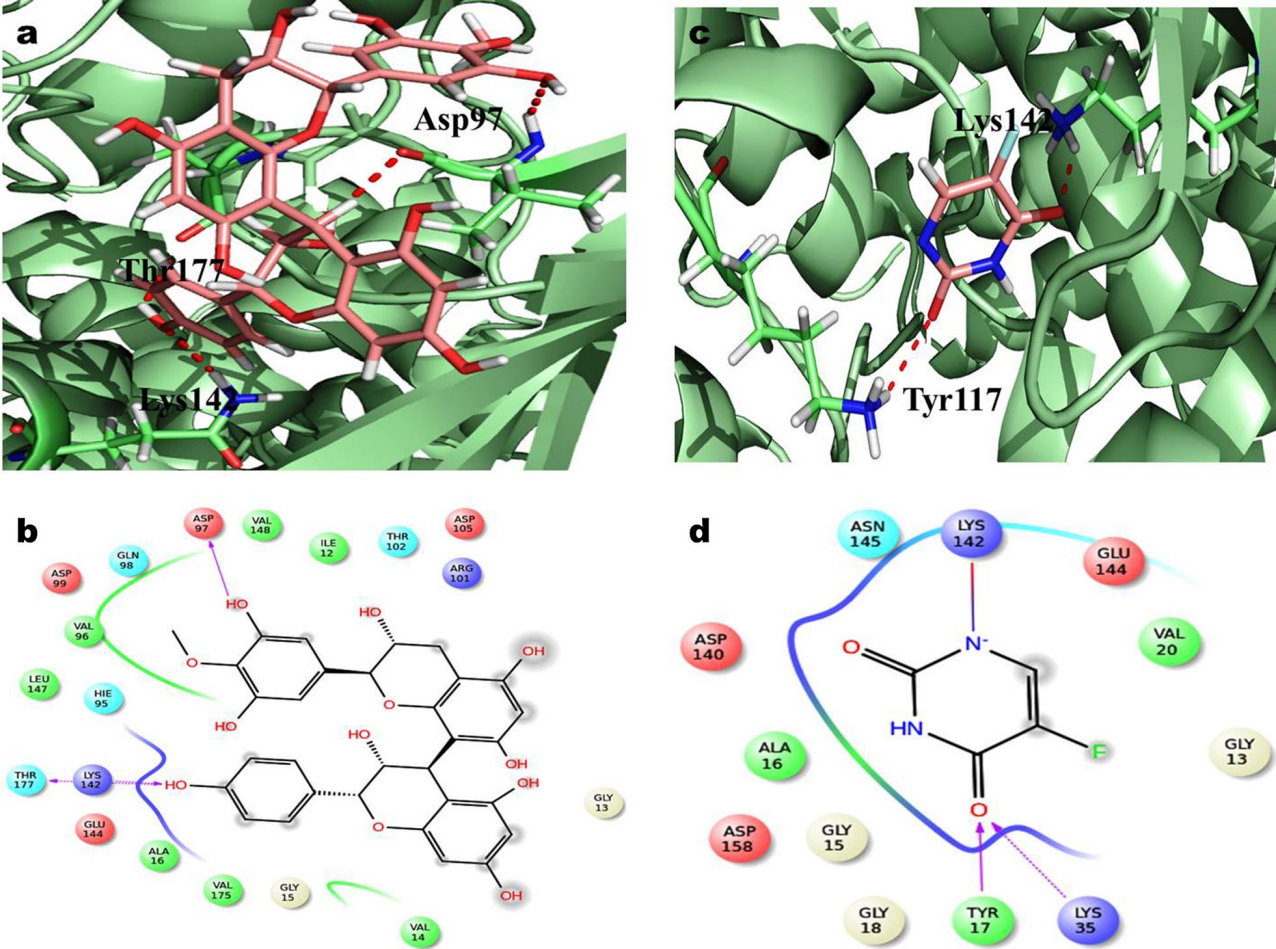
Glide XP docking interaction poses of proanthocyanidin (PAC) and 5-FU with CDK4 **a** PAC, **b** QPLD docking poses of PAC, **c** 5-FU and **d** QPLD docking poses of 5-FU

In CDK2 complex, proanthocyanidin interactions with three hydrogen bond, were examined with Asp86, Asn132 and Gly8 active site remainder using QPLD simulation (Fig. 9). The predicted docking score of QPLD were in − 5.17 kcal/mol. 5-FU complex in the development of two hydrogen bonds with Leu83 in QPLD analysis. Therefore the docking score for 5-FU is found in − 4.47 kcal/mol respectively. The docking examination of QPLD, Proanthocyanidin displayed three hydrogen bond correlations with vital amino acid of Asp97, Thr177 and Lys142 involve as depicted in (Fig. 10). Hence, the 5-FU of QPLD analysis exhibited two hydrogen bond interaction with amino acids Lys142 and Tyr117 active residue of CDK4. The docking score for Proanthocyanidin and 5-FU is determined as − 4.23 kcal/mol and − 4.77 kcal/mol respectively (Table 1).

**Table 1:** Glide XP analysis of proanthocyanidin and 5-fluorouracil

Proteins	XP analysis of docking scores							
	Proanthocyanidin				5-Fluorouracil			
	Docking score	Glide E model energy	H-bonds	Interacting residue	Docking score	Glide E model energy	H-bonds	Interacting residue
BCL-2	− 4.51	− 42.19	5	Arg105, Glu95, Asp99	− 3.72	− 23.67	2	Asp99
BCL-XL	− 5.23	− 65.83	2	Asp107, Ala104	− 4.43	− 20.79	2	Trp1337, Gly138
CDK2	− 5.17	− 60.94	3	Asp86, Asn132, Glu8	− 4.47	− 25.05	2	Leu83
CDK4	− 4.23	− 27.17	3	Asp97, Thr177, Lys142	− 4.77	− 33.76	2	Lys142, Tyr117

### Binding free energy calculation

The obligatory clear force prediction using MM-GBSA recorded in the (Table 2). Proanthocyanidin complex binding with free energies of protein ranged from − 60.02 to − 52.51 kcal/mol of (Bcl2, Bcl-XL). Among them, CDK2 and CDK4 of Proanthocyanidin complex exhibited significant binding free energy (− 64.85 and − 62.71 kcal/mol). The CDK2 and CDK4 showed the high interaction with proanthocyanidin, when compared to the targeted apoptotic proteins like Bcl2 and Bcl-XL. In the condition of required complimentary intensity and the extensive energy donor were found as van der Waals (∆G_vdw_), Coulomb transmission (∆G_Coulomb_) with lipophilic strength (∆G_solLipo_) it enhances the required attraction of proanthocyanidin with the connective pocket of proteins Table 3. An accession in the hydrogen bond prediction the non-bonded prediction more important function in stability of protein–ligand complexes.

**Table 2:** Binding free energy calculation of protein-proanthocyanidin and 5-FU complex using MM/GBSA method

Protein	Binding free energy							
	Proanthocyanidin				5-Fluorouracil			
	∆G_Coulomb_^a^	∆G_vdw_^b^	∆G_solLipo_^c^	∆G_bind_^d^	∆G_Coulomb_^a^	∆G_vdw_^b^	∆G_solLipo_^c^	∆G_bind_^d^
BCL-2	− 27.27	− 39.34	− 28.89	− 60.02	− 16.92	− 9.57	− 1.98	− 23.49
BCL-XL	− 30.80	− 35.47	− 30.26	− 52.51	− 5.58	− 39.14	− 51.04	− 77.35
CDK2	− 13.06	− 45.88	− 24.29	− 64.85	− 13.06	− 45.88	− 24.29	− 64.85
CDK4	− 32.14	− 24.90	− 37.40	− 62.71	− 6.4	− 19.22	− 3.45	− 15.66

All the energy values in kilocalories per mole (kcal/mol)

^a^Contribution to the free energy of binding from the Coulomb energy

^b^Contribution to the free energy of binding from the van der Waals energy

^c^Contribution to the free energy of binding from the lipophilic energy

^d^Free energy of binding

**Table 3:** Proteins chosen for docking with proanthocyanidin

S. no	Name of the proteins	PDB ID
1	BCL2	2W3L
2	BCLXL	2YXJ
3	CDK2	5IEY
4	CDK4	2W96

In modern approaches in computational system have enabled virtual screening of drug discovery [26]. The amino acid Leu83 and Asp146 in the water-repellent section as a play vital function in regulating with CDK2/cyclin B complex activity [27]. This effect has been approved with the previous statement to evaluate the intensity of CDK2 as a pharmaceutical objective for tumor treatment. The hydrogen bond structure with cyclin B/CDK2 and dockings core indicates that proanthocyanidin has able to suppress the properties of CDK2 protein in cell division are productively than 5-FU. Regarding, the crucial amino acids His95 and Val96 act as the decisive role in CDK4 activation [28]. The post-scoring approach were evaluated by using the docking complexes. MM/GBSA obtain the free energy computation process, which helps a consolidation of molecular logistics intensity and constant resolver modes. In the terms of hydrogen and non-hydrogen bond interactions with protein–ligand complex, such as van der Waals, electrostatic energies, polar and non-polar destruction free forces with the supplement form of entropy [29]. Thus, these proteins were considered as receptors to explore the anticancerous activity of selected polyphenolic compounds. It is observed that the binding affinity of PAC was higher with BCL-XL (− 5.23 kcal/mol) and CDK2 (− 5.17 kcal/mol), when compared to the other proteins of BCL2 and CDK4. Interestingly, these results are in agreement with the in vitro and in vivo analyses, which indicated that PAC is having a better inhibition activity of these compounds is found to be higher when compared to 5-FU. In our experience, this is the first report to explore the anti-cancer activity of PAC against Colon cancer.

## Conclusions

The present study displayed the therapeutic activity of PAC as an effective anticancer agent on colon cancer (HT-29) cells. The cell growth and AO/EtBr staining exposed the linguistic difference of PAC compound promotes the apoptotic cells with chromatin condensation and membrane blebbing in HT-29 cells. The cell cycle arrest G2/M phases and percentage of apoptotic cells was confirmed by the dose-dependent manner and apoptotic cell death. Hence we suggest that this flavonoid compound can be considered as an effective anticancerous promoter against human colon cancer cells.
